# Factors associated with patient visits to the emergency department for asthma therapy in Pakistan

**DOI:** 10.1186/s12930-016-0026-y

**Published:** 2016-02-01

**Authors:** Muhammad Bilal, Abdul Haseeb, Mohammad Hassaan Khan, Muhammad Saad, Sapna Devi, Mohammad Hussham Arshad, Anusha Alam, Abdullah Muneer Wagley, Khawaja Muhammad Ammar Ali Javed

**Affiliations:** Dow University Of Health Sciences, Karachi, Pakistan; Aga Khan University Hospital, Karachi, Pakistan; Jinnah Postgraduate Medical Centre, Karachi, Pakistan; Ziauddin University and Hospital, Karachi, Pakistan; Department of Biological Sciences, The Lyceum, Karachi, Pakistan; Department of Biological Sciences, Karachi Grammar School, Karachi, Pakistan; Department of Biological Sciences, Cedar College, Karachi, Pakistan

**Keywords:** Emergency department, Asthma, Inhaled corticosteroids, Pakistan

## Abstract

**Background:**

Acute asthma is a chronic condition affecting people of all ages around the world and hence, is one of the leading causes of emergency department (ED) visits and hospital admissions globally. Most of them are related to poor patient practices and a weak healthcare system. The aim of our study was to assess the reasons for the increased usage of the ED by asthmatic patients in Pakistan.

**Methods:**

A cross-sectional study was conducted on 600 asthmatic patients reporting to the ED of Civil Hospital Karachi over a 6-month period. The consenting patients were given a questionnaire to fill and the following data was collected: demographic information, duration of the disease, medications prescribed the, frequency of and reasons for outpatient clinic and ED visits for issues related to asthma.

**Result:**

According to our results most of the participants visited the ED to obtain a nebulized bronchodilator (90 %) or oxygen (79.5 %). Moreover, 44.8 % of the people visited the ED to get treatment without any delay and 24.0 % considered that the severity of asthma does not allow the patient to wait for clinic visits. Strikingly, 92.8 % claimed that inhaled corticosteroid therapy treatment should be stopped when patients feel better. Irregular follow ups with clinics, low education about asthma and an education level higher than a Bachelors degree were the most important factors associated with three or more ED visits per year, p values = 0.0289, 0.0110 and 0.0150 respectively.

**Conclusion:**

This study identifies several preventable risk factors responsible for recurrent visits to the ED by asthmatic patients in Pakistan.

## Background

Asthma is defined as a chronic inflammatory disorder of the airways that leads to symptoms like wheezing, coughing, breathlessness and tightness of chest, especially at night or in the morning [1]. It is a common condition that affects people of all ages throughout the world. According to the World Health Organization (WHO), 235 million people suffered from asthma worldwide. However, recent figures from the Global Asthma Report 2014 show that the figure may have risen to 334 million. Asthma is the most common chronic disease amongst children according to the WHO [2]. Globally, asthma has affected 5–10 % of entire population; which has increased greatly in last 20 years [2]. In 2010, its prevalence reached to just over seven million. It was also responsible for 640,000 Emergency department (ED) visits, 6.7 million private office visits, and 157,000 hospital admissions in 2007 [3]. There is not much data available overall for Pakistan but some figures are present in relation to the child population. Its prevalence in the pediatric population in Pakistan has increased from 5 to 20 % in the last 20 years [4].

Acute asthma is one of the leading causes of ED visits and hospital admissions globally [5–7]. Previous studies showed that there were an estimated two million asthma-related ED visits and 480,000 asthma-related hospitalizations in the United States in 2009 alone, with an annual cost of approximately $56 billion [8–10]. There are many causes for the large number of ED visits by asthmatic patients. Some of the major ones are duration of symptoms, poor adherence to medications, lack of awareness about the disease, low socioeconomic status, previous hospitalizations or ED consultations, lack of parental confidence in the medications, allergen exposure, lack of health insurance, single-parent and crowded families [11–16].

The increased use of the ED as first-choice of care by asthmatic patients’ needs to be discouraged because they are an unnecessary and preventable burden on resources that could be better utilized elsewhere. It is therefore important to identify the factors and problems in our healthcare system that are responsible for this trend so that we can address them and save valuable resources. The aim of our study is to assess the reasons for the increased usage of the ED by asthmatic patients in Pakistan.

## Methodology

This cross-sectional study was conducted from September 2014 to February 2015 on 600 patients reporting to the ED of Civil Hospital Karachi. It was approved by the Institutional Review Board of Dow University of Health Sciences (DUHS). The inclusion criterion was such that patients with a reported diagnosis of asthma and on prescribed inhaled corticosteroids (ICS) for a minimum of 5 months were recruited for the study. Patients not having a reported diagnosis of asthma by a physician, not on ICS therapy or suffering from any illness that they were seeking medical care for or other chronic respiratory illnesses, like chronic obstructive pulmonary diseases (COPD), were omitted from the study. The consenting patients were given a questionnaire to fill in the ED by the co-investigators. It had questions pertaining to their demographic information and socioeconomic status. Also, their education about the disease was evaluated from the following questions—duration of the disease, the medications prescribed and adherence to them and the method of use of inhaler devices. They were also asked about the frequency of outpatient clinic and ED visits for issues related to asthma. Their files were cross-checked to compare the information that they provided with documented visits and confirm its validity.

The data was entered using Social Sciences (SPSS) software program for Windows (version 19.0) and the same software was used for statistical analysis. Descriptive statistics, such as means, standard deviations, or median were employed to outline age and duration of asthma disease. Percentages were also used to indicate gender, ICS use, follow up with clinics, general education level, education about medications, education about asthma, and reasons for visiting the ED. Moreover, in order to compare the distributions of asthma disease duration across number of asthma-related ED visits (<3 versus ≥3), Mann–Whitney test was applied. The associations between gender, ICS use, follow up with clinics, general education level, education about medications, and education about asthma across asthma-related ED visits were determined by Chi square test. Multiple logistic models were also employed to assess the risk factors that were linked with three or more asthma-related ED visits. p values less than 0.05 were considered significant. The odds ratios (ORs) with 95 % CIs were further considered to report the strength of these associations.

## Results

A total of six hundred (N = 600) asthmatic patients were selected by non-probability convenience sampling in this cross sectional study. Out of 600 participants, 186 (31.0 %) were males and 414 (69.0 %) patients were females. Table 1 depicts the demographic and clinical characteristics of study participants. The mean patients’ age was 47.1 ± 18.7 years, and the mean duration of asthma illness was 147.67 ± 123.19 weeks. Moreover, only 30 % (N = 180) of the patients had regular follow up with the physician, while 70 % (N = 420) of the patients did not have any follow up after their diagnosis of asthma. Three hundred and ninety (65.9) patients did not have any formal education about asthma, whereas 70.4 % (N = 422) were uneducated with regards to usage of devices and medications. Of 210 patients who received education about asthma as a disease, 55.0 % acquired it from physicians, 13.8 % from asthma educators and the rest from pharmacists. Similarly, when patients were enquired about the reason for ED visit, it was found that 90.0 % visited to obtain a nebulised bronchodilator, 79.5 % to obtain oxygen, 44.8 % to get treatment without any delay while 24.0 % considered that severity of asthma does not allow the patient to wait for clinic visit. Strikingly, 83.0 % of the participants were unaware about the factors that trigger symptoms of asthma and 92.8 % claimed that ICS treatment should be stopped when patients feel better. Table 2 depicts knowledge about asthma management and reasons for visiting the ED.

**Table 1:** Demographic and clinical characteristics of study participants

Demographic characteristics	N	%
Age (years) Mean ± SD	47.1 ± 18.7	
Duration of illness in weeks (Mean ± SD)	147.67 ± 123.19	
Gender		
Male	186	31.0
Female	414	69.0
Level of education		
Matriculation (grade 10)	93	15.5
Intermediate (grade 12)	89	14.8
Bachelors	60	10.0
Masters	39	6.5
No education	319	53.2
Profession		
Labor	180	30.0
Businessman	64	10.7
Government employee	122	20.3
Private employee	142	23.6
Housewife	60	10.0
Retired	32	5.4
Follow up regularly with doctor	180	30.0
Follow-up clinic		
PHC/Family Medicine	129	21.5
Pulmonary	31	5.2
Internal Medicine	20	3.3
No follow-up	420	70.0
No education about asthma	390	65.0
No education about medication (devices)	422	70.4
ED visits		
<3	399	66.5
≥3	201	33.5

All percentage rounded to one decimal

**Table 2:** Knowledge about asthma management and reasons for visiting ED department

Variable	N	%
Reason for ED visit		
Visit ED primarily to obtain a bronchodilator	540	90.0
Visit ED to obtain oxygen	477	79.5
The severity of asthma doesn’t allow the patient to wait for a clinic visit	144	24.0
Belief that the patient is treated faster in the ED	153	25.5
The ED is available 24 h a day	243	40.5
The patient treated directly without delay	269	44.8
Medication given as nebulizer at ED is more useful	363	60.5
Knowledge about asthma management		
Take bronchodilator to relieve symptoms only	534	89.0
Stop ICS therapy when feel better	557	92.8
Believe long term use of inhaler unsafe	183	30.5
Believe continues use of inhaler cause dependence	244	40.7
Believe asthma therapy use its effect overtime	200	33.3
Does not know what trigger asthma symptoms	498	83.0
Does not know what should do during asthma attack	215	35.8

All percentage rounded to one decimal

Furthermore, asthma-related ED visits were stratified on the basis of whether the asthma patient visited the ED thrice or more for asthma treatment. Table 3 illustrates the relationships between three and more asthma-related ED visits and the patient’s general education level, education about asthma, ISC and education about asthma medications. Those who were not educated about asthma were more likely to visit the ED because of asthma than those who had been educated about asthma (41.5 versus 31.0 %, p value = 0.011). Surprisingly, those who were more educated about asthma medications and had an education level of a Bachelors degree or greater, exhibited more number of visits to the ED for the treatment of asthma (51.8 versus 35.0 %, p value = 0.017; 56.9 versus 34.8 %, p value = 0.023). Besides, it was also revealed that a relationship between patient needing oxygen for asthma therapy and three or more ED visits also exists (55.5 versus 23.92 %, p value = 0.0118). However, there was no correlation between visiting ED primarily to obtain a bronchodilator and three or more ED visits to treat asthma (33.4 versus 41.8 %, p value = 0.407).

**Table 3:** Association between asthma-related ED visits and demographic and clinical characteristics

Variable	Levels	<3 visits (*n * = 399)	≥3 visits (*n* = 201)	*p* value
Gender	% Male	62.5	37.5	0.532
	Female	64.0	36.0	
Regular ICS use	Yes	63.8	36.2	0.284
	No	61.7	38.3	
Follow up with clinics	Yes	65.5	34.5	0.366
	No	62.3	37.7	
Education level	Intermediate (grade 12) or less	65.2	34.8	0.023^a^
	Bachelors or high	43.1	56.9	
Educated about medication	Yes	48.2	51.8	0.017^a^
	No	65.0	35.0	
Educated about asthma	Yes	69.0	31.0	0.011^a^
	No	58.5	41.5	

^a^The Chi square statistic is significant at the 0.05 level. All percentage rounded to one decimal

Mann–Whitney test shows there was no connection between the duration of the disease and the number of ED visits (p  =  0.546). An education level higher than a Bachelors degree (p value  =  0.0150) and irregular follow up with clinics (p value  =  0.0289) were highly associated with three or more asthma-related ED visits, after being controlled for gender, ICS use, general education level, education about medications, and education about asthma. Our study also indicated that patients with a Bachelors degree or higher were twice as more likely to visit the ED than the patients with Intermediate (grade 12) education or less than it (OR: 2.176; 95 % CI: 1.569, 2.783). Moreover, this finding is consistent with patients having education about medications (OR: 1.966; 95 % CI: 1.435, 2.497). It was further revealed that patients who were not educated about asthma also had greater number of visits to the ED for the treatment of their condition (OR: 1.586; 95 % CI: 1.270, 1.902). Table 4 depicts the odd ratios and 95 % CIs for the risk factors associated with three or more asthma-related ED visits.

**Table 4:** Odd ratios and 95 % CIs for the risk factors associated with three or more asthma related ED visit

Variable	Levels	Estimate	p value	OR		95 % CI
Intercept		−0.2767	0.4433			
Age		0.00364	0.6066	1.015	0.876	1.154
Gender	Female	0.0706	0.5397	1.126	0.923	1.329
Regular ICS use	No	0.0532	0.7567	1.153	0.965	1.341
Follow up with clinics	No	−0.2244	0.0289^a^	0.770	0.656	0.884
Education level	University	0.4189	0.0150^a^	2.176	1.569	2.783
Educated about medication	Yes	0.0810	0.0442^a^	1.966	1.435	2.497
Education about asthma	No	0.1943	0.0397^a^	1.586	1.270	1.902

^a^Wald Chi square statistic is significant at the 0.05 level

## Discussion

The results of this study showed that a large percentage of the Pakistani population used the ED as a first-choice option instead of keeping follow up appointments with physicians. These findings are in line with previous studies conducted in Saudi Arabia, New Zealand and the United States [17–19]. We also found that patients do not receive much education about the disease they are suffering from and the methods of controlling or treating it. It was evident that 65 % of the population was unaware about asthma as a disease while around 70 % were uninformed about the medications and devices, such as inhalers. These numbers are greater than those of AL-Jahdali et al., who found that around 52 % of people were uneducated regarding asthma and approximately 41 % about the medications. There are multiple reasons for the high illiteracy rate related to asthma education. It is due to a lack of properly trained and educated staff in the tertiary care hospitals and the low level of education of the patients. Previous studies have suggested that the healthcare workers are untrained and lack the basic knowledge regarding the use of inhalation devices, let alone the more modern methods, hence they are ill-positioned to guide patients on how to use their medicine [20–23]. It has been shown that good asthma control is related to patient self-management so it is important to ensure that the patient has all the required information to manage his condition [24–27]. Moreover, only 13.8 % of study population received education from asthma educators which could be further linked to a limited number of trained staff in our health care system. Consequently, the number of patients visiting the ED for the treatment of asthma exacerbations increases greatly.

Furthermore, these results highlight major flaws in our healthcare system and show that there is a desperate need for better training of doctors, better education of and easier access to clinics for patients and generalized awareness programs related to asthma for the community. A huge problem that we do face is that asthma patients do not respond to follow ups with the figure an astounding 70 %. Out of the remaining 30 % who do, around 22 % visit the primary care clinic where the guidance is unsatisfactory as indicated by work of Abudahish A et al. [28] This study conducted in Saudi Arabia found that 40 % of people did not attend any follow up appointments and of the remaining who did, around 46 % attended the primary care clinic [17]. These numbers point out a huge flaw in the primary healthcare system, with asthma management not quite up to scratch. Further results from our study showed that there was widespread misconception about using the ED amongst patients with 90 % using it to get a bronchodilator and 80 % to get oxygen. These values were comparatively less in the study carried out by AL-Jahdali et al., around 87 and 75 %. These visits were unnecessary, as these medications could have been easily obtained from normal clinics too, and utilized precious resources that could have been put to better use, for more critically ill patients.

Moreover, it is a well-known fact that individuals with higher education level have a higher socioeconomic levels. As a result they have a better health status due to the reason that they have greater access to healthcare services. Interestingly, we found that the more educated patients reported three or more visits to the ED, results that were consistent with a previous study in Saudi Arabia [17]. However, unlike that study, patients with knowledge about the medication reported more than three visits to the ED [17]. These findings could be explained by the fact that the patients with asthma experience high levels of anxiety, especially during an exacerbation of their symptoms, which may interpret the increased use of ED services, regardless of sociodemographic factors.

According to our understanding, this study is a first of its kind to be carried out in Pakistan. It was a very extensive process involving direct communication with the patients and then confirming the information received with the patient files. It is an important step in the right direction because it identifies the frailties in the healthcare system. Further studies are required in this field so that we can improve the framework for asthma care in this country.

## Limitations

This study was carried out only in one hospital so our sample size was a bit restricted and these results do not tell the story of the situation in the whole country. The patients that we got were mostly from a low socioeconomic and relatively illiterate background so we cannot generalize these results to other patients who might have a different knowledge of asthma. Other factors, like access to primary care, may have affected the recurrent use of the ED but we could not take them into consideration due to the unavailability of relevant data. The data was also collected in written form via questionnaires and it might have affected the results as the patients, some of whom were not very educated, might not have comprehended some of the questions. Therefore, verbal questioning could be added for better results. We could also only quantify the education received by the patients regarding asthma and not really judge the quality of it. We also did not take into account other environmental factors in the daily routine of the patient responsible for exacerbation of the disease, leading to ED visits. Furthermore, we could not accurately judge the socioeconomic status of the patients because we only used profession and level of education as indicators. Also, a previous study has indicated that people tend to downplay their level of economic disadvantage when reporting it [29].

## Conclusion

This study identified several factors responsible for recurrent visits to the ED for asthma care. The important ones were patient misconceptions about the use of the ED in treating asthma, the lack of education regarding asthma, the lack of adherence to follow up appointments with exclusive asthma clinics and the decreased adherence to asthma medication. These factors need to be addressed by healthcare workers and there needs to be an overhaul at the highest level in order to improve the healthcare system. The government also needs to focus more on the education part in primary healthcare rather than giving all their attention to the tertiary sector. It is the only way to decrease the load on the tertiary care system and prevent the drainage of important resources.

## Abbreviations

ED: emergency department
ICS: inhaled corticosteroid therapy
COPD: chronic obstructive pulmonary disease
